# Biased brain and behavioral responses towards kin in males of a communally breeding species

**DOI:** 10.1038/s41598-023-44257-6

**Published:** 2023-10-09

**Authors:** Brandon A. Fricker, Deborah Ho, Ashley W. Seifert, Aubrey M. Kelly

**Affiliations:** 1 Department of Psychology, Emory University, 36 Eagle Row, Atlanta, GA 30322 USA; 2 Department of Biology, University of Kentucky, 101 Morgan Building, Lexington, KY 40506 USA

**Keywords:** Social behaviour, Social neuroscience

## Abstract

In complex social environments, individuals may interact with not only novel and familiar conspecifics but also kin and non-kin. The ability to distinguish between conspecific identities is crucial for most animals, yet how the brain processes conspecific type and how animals may alter behavior accordingly is not well known. We examined whether the communally breeding spiny mouse (*Acomys cahirinus*) responds differently to conspecifics that vary in novelty and kinship. In a group interaction test, we found that males can distinguish novel kin from novel non-kin, and preferentially spend time with novel kin over familiar kin and novel non-kin. To determine whether kinship and novelty status are differentially represented in the brain, we conducted immediate early gene tests, which revealed the dorsal, but not ventral, lateral septum differentially processes kinship. Neither region differentially processes social novelty. Further, males did not exhibit differences in prosocial behavior toward novel and familiar conspecifics but exhibited more prosocial behavior with novel kin than novel non-kin. These results suggest that communally breeding species may have evolved specialized neural circuitry to facilitate a bias to be more affiliative with kin, regardless of whether they are novel or familiar, potentially to promote prosocial behaviors, thereby facilitating group cohesion.

## Introduction

Large group-living is phylogenetically widespread and is advantageous for numerous species, including insects^1,2^, birds^3,4^, and mammals^5–7^, providing benefits such as collective traveling^8,9^, more effective homeostatic regulation^10,11^, predation reduction^12–14^, and enhanced offspring survival^15–17^. Large group-living inherently yields complex social environments, such that conspecifics an individual encounters will vary in the degree of novelty (strangers, acquainted, etc.) and their kinship status to a much greater extent than for small group-living species. In such dynamic environments, accurately recognizing whether an individual is familiar or related is likely highly adaptive and promoted through unique sensory cues^18,19^ that facilitate context-appropriate behavioral choices^20–23^. Indeed, social recognition likely promotes successful group identification^22,24^, accurate navigation of social hierarchies^25,26^, and incest avoidance^1,27^.

Communal breeding is a social structure with a highly complex social environment. Communal breeders, like Degus, *Octodon degus*^7,28^, can have several breeding pairs of mixed relation, and most group members engage in parenting^29^. Further, there is high group member turnover in Degus, resulting in a frequent influx of social novelty^28^. Because there are varying degrees of genetic relationships in communally breeding groups, the ability to distinguish between kin and non-kin, regardless of familiarity, is especially important to avoid incest and promote navigation of the social environment in an optimal manner. For example, individuals that disperse to a neighboring group that contains novel, older relatives would need to rely on kin recognition more than familiarity for accurate social recognition of kin.

In large group-living, communally breeding species, social recognition may be dampened to promote indiscriminate parental care among the entire population, thereby facilitating group cohesion. However, this strategy would likely result in high rates of incest and unviable offspring. Alternatively, then, social recognition abilities may indeed be high, but social motivation systems may have evolved to deprioritize genetic relation specifically for raising offspring but not for mating. The conflict between high rates of socially novel encounters and indiscriminate parental care raises the question as to whether the ability to distinguish between kin and non-kin or between novel and familiar individuals is dampened in communally breeding species. Further, whether these distinct types of social recognition are modulated via similar mechanisms, to our knowledge, remains unknown.

The spiny mouse, *Acomys cahirinus,* is a communally breeding species primarily found in the deserts of Africa, the Middle East, and Southeast Asia^30^. Spiny mice naturally live in large groups^30^; in the lab they are highly prosocial and prefer affiliating with large groups over small groups^5^. Additionally, spiny mice readily accept a newcomer into an established group^31^. Thus, spiny mice’s social structure provides a particularly complex and challenging environment for social recognition. This naturally complex social landscape allows us to explore preferences and recognition abilities for distinguishing between kin and non-kin, while controlling for familiarity and novelty, in ethologically valid ways that are often challenging in other species. Thus far, spiny mice have been shown to successfully identify direct and cross-fostered littermates through olfactory cues and phenotype matching based on the female they nursed from^19^, and we have previously shown they accurately recognize a new conspecific after repeated exposure to a different individual in a social recognition test^5^. However, how novelty and kinship may interact to influence behavior in a group and whether the neural underpinnings of these distinct types of social recognition differ have yet to be explored.

One potential brain region contributing to the neural underpinnings of social recognition in spiny mice may be the lateral septum (LS). In recent years, the LS has become an increasingly examined region for social behaviors, such as aggression^32^, social exploration^33^, flocking^3^, prosocial behavior^34^, and social recognition^35,36^. While studies have examined contributions of the LS to social novelty recognition, fewer studies have specifically sought to determine whether the LS also differentiates kin from non-kin, especially while controlling for novelty^33,37,38^. Clemens et al.^36^ showed that lesioning of the LS disrupts a preference for siblings and identified a neural topographic mapping of kinship in the LS of Long Evans rats. However, while these results position the LS as a promising region for regulating social recognition, and specifically kin recognition, in spiny mice, the study did not control for the familiarity of littermates compared to novel non-siblings. Thus, it is unclear not only if the LS holds a similar role for kin recognition in spiny mice as it does for a highly selected strain of domestic rat with low levels of social motivation^39^, but also whether the LS differentiates between novelty and familiarity in this species.

Here we examined whether spiny mice appear to recognize and behave differently based on both the novelty and the kinship status (kin versus non-kin) of individuals they interact with in group and dyadic social contexts. Because we previously found that spiny mice exhibit no preference for affiliating with or investigating novel or familiar conspecifics, we hypothesized their behavior would not differ during social interactions with novel and familiar non-kin conspecifics but would differ between novel kin and novel non-kin. Further, to determine if context-specific social recognition occurs via processing in the same brain region, we used immediate early gene (IEG) studies to examine whether the LS distinguishes novelty and familiarity as well as kinship status. Because of previous findings revealing a topographic map for kinship in rats within the LS^36^, demonstrating how subregions within the LS can have separate functions^32,40^, we hypothesized that the LS would differentially process exposure to novel kin versus novel non-kin, potentially in a spatially distributed manner. Lastly, because communal breeders frequently interact with novel and familiar conspecifics, and because spiny mice show no preference for affiliating with or investigating novel or familiar conspecifics^5^, we hypothesized that the LS would not show differentiated responses between novel and familiar non-kin.

## Results

### Male spiny mice differentiate between kinship and familiarity and preferentially affiliate with novel kin in a novel, same-sex group

**Figure 1:**
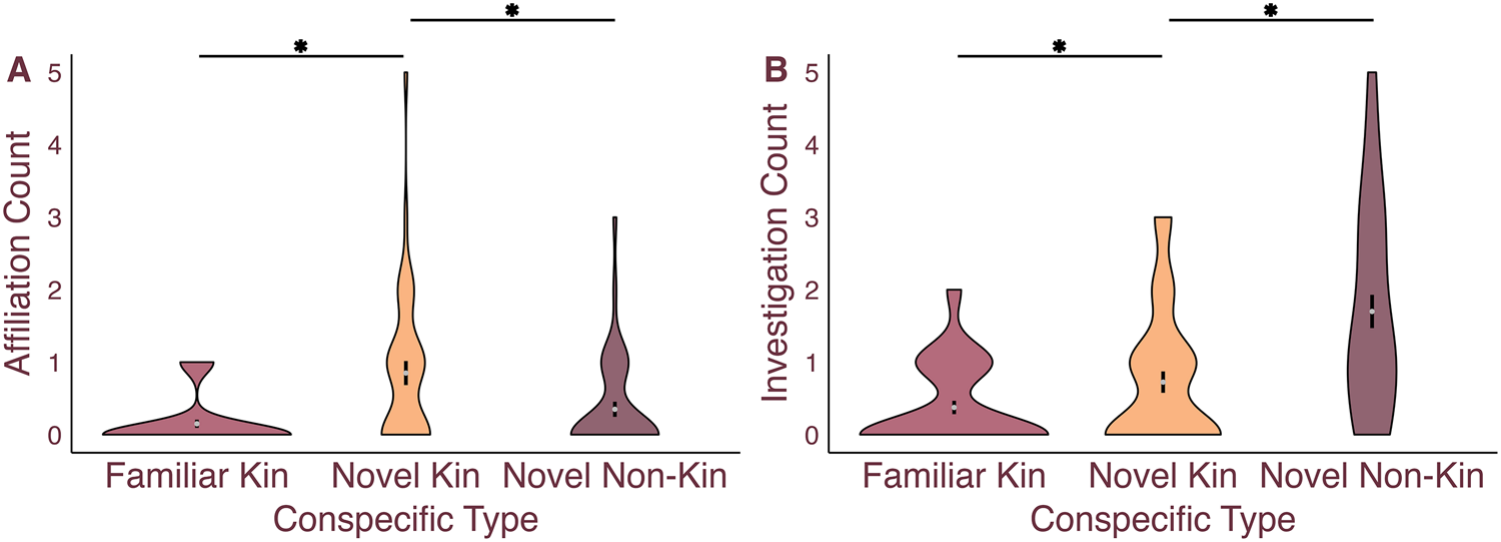
Male spiny mice differentially investigated and affiliated with peers based on conspecific type in a group interaction test. Male spiny mice mean frequencies represented in a violin plot with the mean ($\pm$ SEM) represented for (**A**) affiliation and (**B**) investigation during a group social interaction. Spiny mice spent significantly more time investigating novel kin (orange) over familiar kin (wine) and novel non-kin (plum) and preferred to affiliate with novel kin. Dots represent individual data. *Indicate P ≤ 0.05.

Here we aimed to examine whether spiny mice recognize and behave differently with conspecifics based on the novelty and kinship status of the individuals they interact with within a group. 5 groups of 8 male spiny mice were placed into a novel arena for a 1-h group interaction test. Each subject was familiar with only one other conspecific—their sibling and cagemate. For each subject, 2 others were novel kin, and the remaining 4 were novel non-kin. Novel kin were at least 2 litters removed from the subject spiny mice. Time spent investigating and positively affiliating with each stimulus type (familiar kin, novel non-kin, and novel kin) were sampled for each subject every 5 min. A Poisson generalized linear mixed model (GLMM) analysis with conspecific type as a fixed factor, group as a random factor, and subject as a random factor to account for within-subject testing found that the frequencies of investigation (X^2^_(2,120)_ = 35.281, p < 0.001; Fig. 1A) differed based on stimulus type. Specifically, Sidak-corrected post hoc analyses revealed that male spiny mice investigated novel non-kin more than familiar kin (N = 40, MD = 1.24, p < 0.001) and novel kin (N = 40, MD = 0.91, p < 0.001). Additionally, a Poisson GLMM analysis yielded a main effect of affiliation frequencies (X^2^_(2,120)_ = 19.423, p < 0.001; Fig. 1B), with Sidak-corrected post hoc analyses showing that males affiliated more with novel kin than novel non-kin (N = 40, MD = 0.48, p = 0.012) and more with novel kin than familiar kin (N = 40, MD = 0.68, p < 0.001). These results suggest spiny mice accurately differentiate between kin and non-kin regardless of the conspecific’s novelty status and that spiny mice opt to investigate novel non-kin more; this may be to gather more information about unknown individuals. Further, affiliation preferences upon forming a new group may be biased toward interacting with novel kin.

The findings from the group social interaction test show that male spiny mice discriminate between novel kin, novel non-kin, and familiar kin and can distinguish novel kin from novel non-kin, even if they preferentially affiliate with novel kin. These results suggest kinship status and novelty may interact in a complex and nuanced way when making social decisions in the communally breeding spiny mouse and begs the question whether distinct types of social discrimination are differentially reflected in brain regions important for social behavior. The LS has recently been proposed as a hub for processing social information^41^ and a recent study in Long Evans rats demonstrated that kin versus non-kin responsive cells may be topographically organized^36^; however, this paper did not control for social novelty. Thus, we next conducted IEG studies to examine whether neurons in the LS differentially process (a) novelty and familiarity when controlling for kinship, as well as (b) kin and non-kin when controlling for novelty. To specifically examine neural responses in the LS, we quantified NeuN-immunoreactive (ir +) cells (a neuronal marker) colocalized with Fos, a marker of neural activity.

### LS NeuN-Fos colocalization does not differentiate between familiar and novel conspecifics

**Figure 2:**
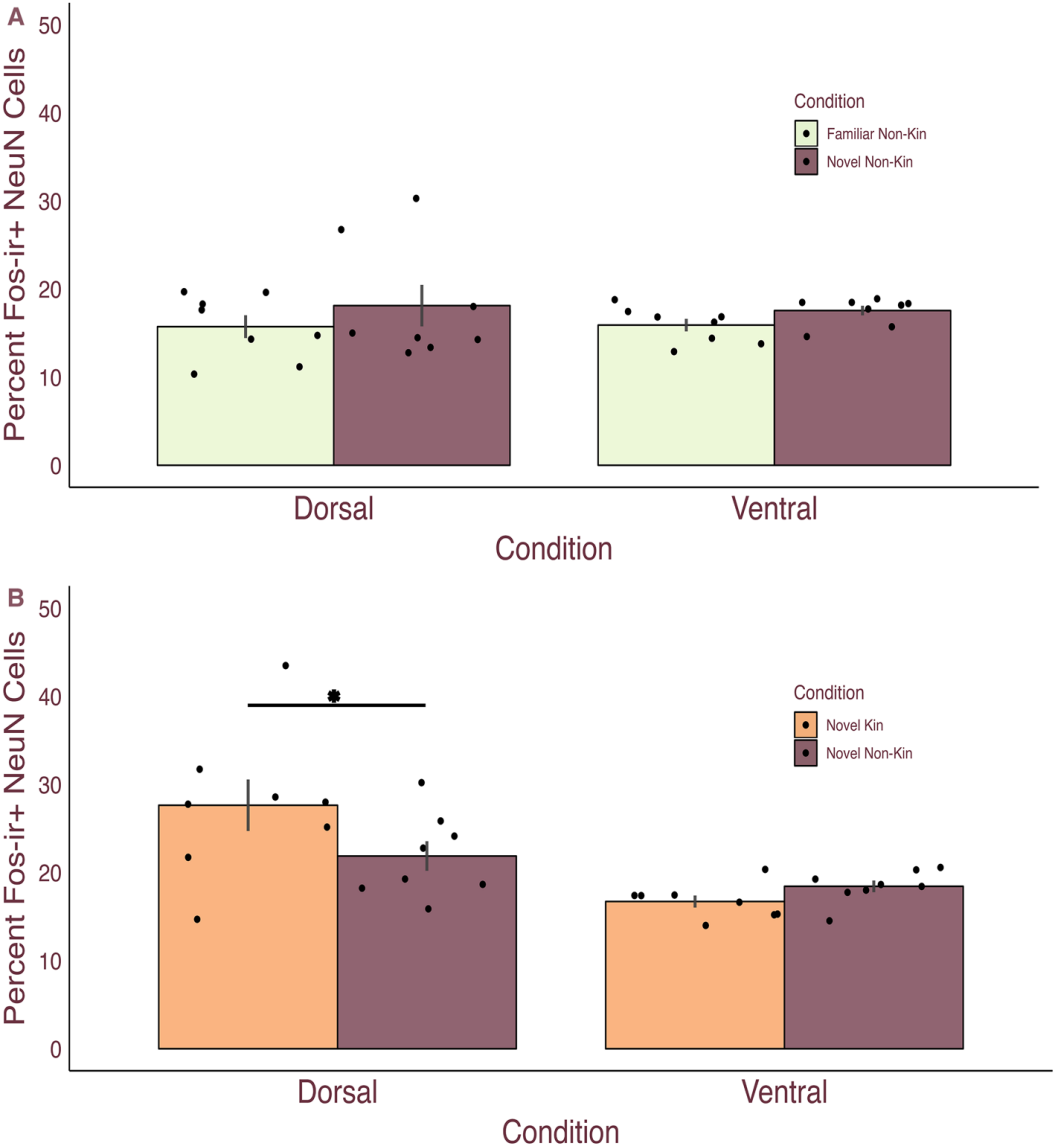
Lateral septum neural responses differed between novel kin and novel non-kin conspecific exposure. (**A**) Male spiny mice mean ($\pm$ SEM) percentage of NeuN-Fos colocalized cells within the dorsal and ventral lateral septum for familiar non-kin (mint) and novel non-kin (plum). Neither region nor condition significantly differed. Dots represent individual data. (**B**) Male spiny mice mean ($\pm$ SEM) percentage of NeuN-Fos colocalized cells within the dorsal and ventral lateral septum (LS) for novel kin (orange) and novel non-kin (plum). Neural responses were greater in response to interactions with novel kin than non-kin in the dorsal, but not ventral LS. Dots represent individual data. *Indicate P ≤ 0.05.

To identify whether neurons in the LS differentially respond to novelty, we conducted an IEG study where 16 male spiny mice were allowed to freely interact with either a same-sex familiar non-kin conspecific or a same-sex novel non-kin conspecific for 30 min. We analyzed the percentage of NeuN-ir + cells that were also Fos-ir + for both the dorsal and ventral LS. A GLM with condition (novel non-kin or familiar non-kin) and LS region (dorsal or lateral) as fixed factors yielded no main effects for condition (F_(1,28)_ = 2.031, p = 0.165), or region (F_(1,28)_ = 0.018, p = 0.893) nor an interaction (F_(1,28)_ = 0.069, p = 0.795) (Fig. 2A). These results suggest the LS does not show differentiated responses to familiarity when controlling for kinship.

### Spiny mice investigate different areas of the body on novel and familiar conspecifics

Despite a lack of neural response differences within the LS as measured by NeuN-Fos colocalization, behavioral differences during interactions with novel and familiar non-kin may still be present. Therefore, we analyzed behavior from the first 5 min of the IEG study to determine if spiny mice interacted differently with novel and familiar non-kin conspecifics. The overall behavioral breakdown for each condition was compared via Friedman’s test and post hoc Wilcoxon ranked sum tests. For both conditions, time spent engaging in prosocial, aggressive, and non-overt behaviors differed (${\chi }^{2}$(2) = 16, and p < 0.001 for both) (Table S1). Between overt (i.e. interactive) behaviors, spiny mice in both conditions were more prosocial than aggressive (all Z = − 2.521, p = 0.012, r = 0.63) (Table S1).

In addition to overall behavioral breakdowns, because rodents are primarily olfactory communicators, we sought to determine whether differences in investigation time could be detected based on the bodily location of stimulus animal. A Friedman’s test revealed that subjects that interacted with novel non-kin conspecifics did not differentially investigate the head, flank, or rear of stimuli (${\chi }^{2}$(2) = 1.750, p = 0.417). However, subjects that interacted with familiar non-kin stimulus animals differentially investigated distinct areas of the stimulus’ body (${\chi }^{2}$(2) = 7.750, p = 0.021) (Fig. S1). Specifically, after false discovery rate correction on a Wilcoxon Signed Ranks test results, males investigated the flank more than the head of familiar non-kin conspecifics (Z = -2.521, p = 0.036, r = 0.63) (Fig. S1). Although we observed no significant differences in LS responsivity to novel vs. familiar non-kin, our behavioral data suggests that male spiny mice discriminate between novel non-kin and familiar non-kin.

### LS NeuN-Fos colocalization differentiates between novel kin and novel non-kin

We next aimed to determine if neurons within the LS differentially process kinship. We conducted an IEG study where 16 male spiny mice were allowed to freely interact with either a novel kin conspecific or a novel non-kin conspecific for 30 min.

The percentage of NeuN-ir + cells that were also Fos-ir + for both the dorsal and ventral LS was analyzed via a GLM with condition (novel kin versus novel non-kin) and LS region (dorsal or lateral) as fixed factors. While there was no main effect of condition (F_(1,28)_ = 1.334, p = 0.258), we found a main effect of LS region, such that Bonferroni post hoc analysis revealed the dorsal LS had a higher percentage of colocalized cells than the ventral LS (F_(1,28)_ = 16.332, p < 0.001). Additionally, we observed a significant interaction (F_(1,28)_ = 4.573, p = 0.041) with Bonferroni post hoc analysis identifying that the dorsal, but not ventral, LS had a higher percentage of colocalized cells in the novel kin condition (M = 27.65% ) compared to the novel non-kin condition (M = 21.878%; MD = 5.77, SEM = 2.48, p = 0.027, d = 0.69) (Fig. 2B). This finding suggests that the dorsal LS may process kinship status.

### Spiny mice are more prosocial toward novel kin than novel non-kin

**Figure 3:**
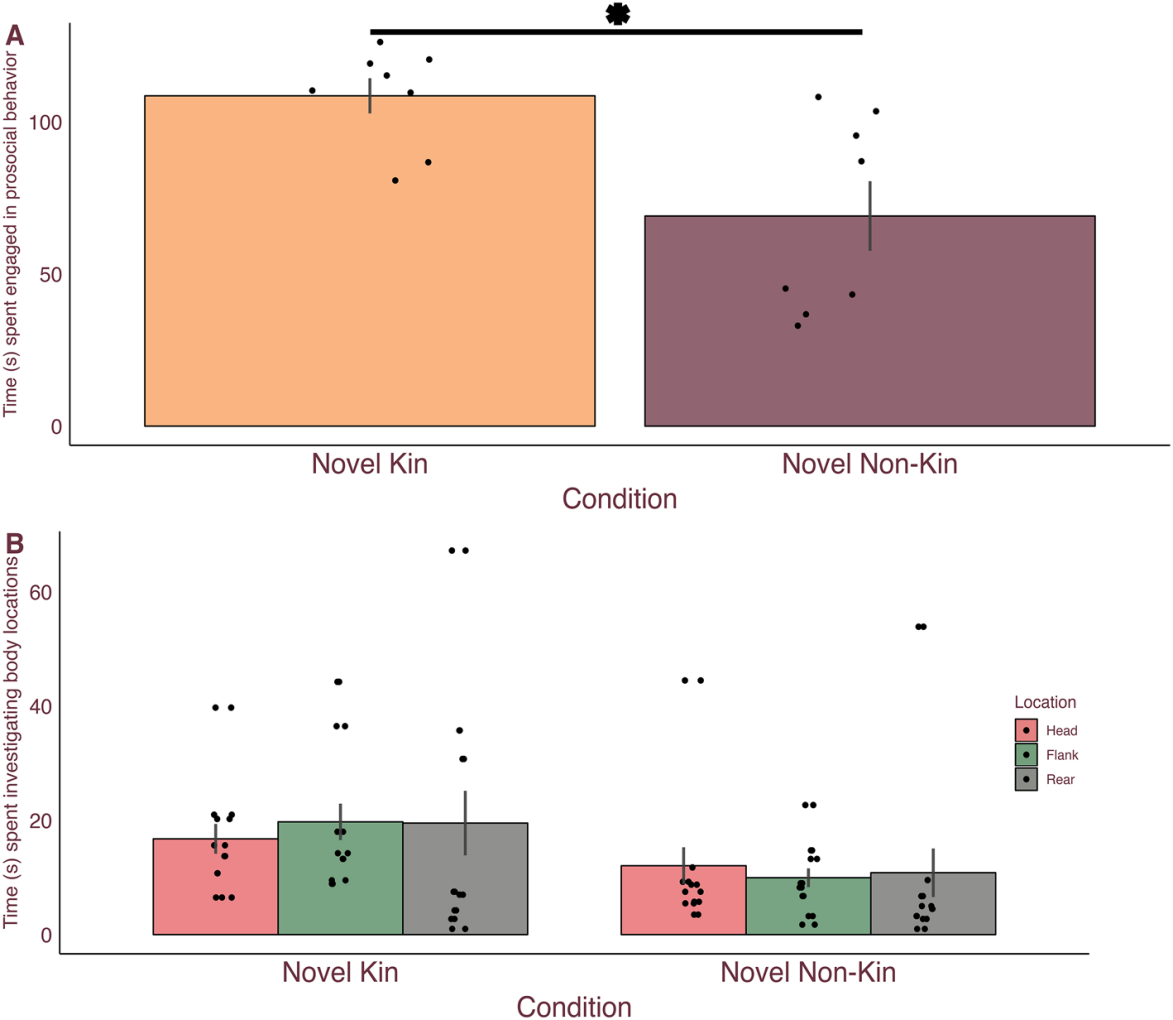
Male spiny mice engaged in more prosocial behavior with novel kin than novel non-kin but did not investigate them differently. Male spiny mice mean ($\pm$ SEM) time in seconds (s) engaged in (**A**) prosocial behavior and (**B**) investigation. Male spiny mice spent more time engaged in prosocial behavior with novel kin (orange) than novel non-kin (plum). Spiny mice displayed a trend towards investigating novel kin more than novel non-kin specifically at the flank (green). Dots represent individual data. *Indicate P ≤ 0.05.

Similar to the previous IEG study, the overall behavioral breakdown for each condition was compared via Friedman’s test and post hoc Wilcoxon ranked sum tests. For both conditions, time spent engaging in prosocial, aggressive, and non-overt behaviors differed (${\chi }^{2}$(2) = 16, and p < 0.001 for both) (Table S2). Between overt (i.e. interactive) behaviors, males in both conditions were more prosocial than aggressive (all Z = − 2.521, p = 0.012, r = 0.63) (Table S2). Between conditions, male spiny mice that interacted with novel kin spent more time engaging in prosocial behaviors (U(n_1_ = 8, n_2_ = 8) = 8, Z = -2.521, p = 0.012, r = 0.63) and less time in non-overt behaviors (U(n_1_ = 8, n_2_ = 8) = 8, Z = -2.521, p = 0.012, r = 0.63) compared to males that interacted with novel non-kin conspecifics (Fig. 3A). Additional analyses revealed no difference in males for their investigation in the novel kin condition compared to the novel non-kin condition (U(n_1_ = 8, n_2_ = 8) = 14, Z = -1.890, p = 0.059, r = 0.50) (Fig. 3B).

### Ventral, but not dorsal, LS neural responsivity relates to behavior

To examine brain-behavior relationships, we conducted Pearson’s correlation analyses between the percentage of NeuN-Fos colocalized cells and investigation as well as time spent engaging in prosocial behavior with stimulus animals. We did not observe any significant brain-behavior correlations in males that were exposed to familiar conspecifics (all p > 0.128). However, we found a positive correlation between percentage of NeuN-Fos colocalization in the ventral LS and total investigation time (r = 0.703, p = 0.052) of novel non-kin. Further, we observed significant correlations between ventral LS NeuN-Fos colocalization and investigation of the flank of stimuli for males exposed to novel non-kin (r = 0.757, p = 0.03; Fig. S2A) as well as those exposed to novel kin (r = 0.727, p = 0.041; Fig. S2B). Together, these findings suggest that the ventral LS may play a particularly important role in investigative behavior of novel conspecifics.

## Discussion

Properly recognizing the kinship and novelty status of individuals is an important component of navigating the social environment, especially for large group-living, communally breeding species that encounter a wide variety of conspecifics. Here we showed that spiny mice alter their behavior in group and dyadic contexts based on the novelty and kinship status of conspecifics. As has been shown for many species (e.g. Ref.^42–44^), we demonstrated that spiny mice recognize novel kin from novel non-kin; additionally, we showed that males differentially investigate bodily locations of conspecifics based on identity. Further, in a novel group interaction and in dyadic interactions, males preferentially affiliate and engage in prosocial behaviors with novel kin over novel non-kin and familiar kin, suggesting that a general drive to affiliate with novel relatives may have evolved to promote group cohesion in communally breeding species. Even though encountering a novel conspecific involves uncertainty and potentially risk, only kinship status was differentially represented in neural responses within the LS of spiny mice. Together these findings suggest that male spiny mice may have evolved specialized neural circuitry to distinguish kin and behave more prosocially toward them, perhaps to facilitate behaviors such as nepotism.

### Affiliation and conspecific identity in groups

For species that live in complex, large groups, differentially affiliating with specific individuals based on their identity may confer distinct benefits related to fitness and survival^45–49^. The affiliative preference for novel kin observed in the present study adds new context to our previous findings in which spiny mice did not show a preference in time spent with novel non-kin or familiar kin in a 2-choice test despite altering their behavior based on novelty in a social recognition test^5^. Together, these findings reinforce that male spiny mice exhibit strong social recognition abilities and are not neophobic and demonstrate that social preferences arise when animals are allowed to freely interact. Recognizing kin regardless of familiarity is likely highly beneficial for avoiding inbreeding. Similar to our findings here, female meerkats can discriminate between odors of kin and non-kin and spend more time investigating scents from related than unrelated novel individuals^50^. After an individual recognizes a novel conspecific as kin, behaving more prosocially toward them may confer fitness benefits. Indeed, affiliation and prosociality with even distantly related kin have likely contributed to the fitness of other large group-living species. For example, meerkats^51–53^ and prairie dogs^54,55^ live in large groups primarily comprised of kin and engage in mobbing and sentinel behavior to protect the group from predators. Favoring kin can thus aid in protection and potentially recruit alloparents for assistance with rearing offspring. Therefore, although animals that live in large groups may affiliate with kin and non-kin, exhibiting a bias to behave more prosocially with kin likely enhances fitness, perhaps without a direct cost to the group as a whole.

### Kinship and prosociality

Dyadic social interactions allow for more detailed analysis of how individuals interact with specific types of conspecifics. Interestingly, spiny mice did not differ in the amount of time they spent engaged in general prosocial, aggressive, or non-overt behaviors between familiar and novel non-kin conspecifics but did spend significantly more time engaged in prosocial behaviors with novel kin versus novel non-kin. Similar to spiny mice, female Cape ground squirrels, *Xerus inauris*, are a cooperatively breeding species and show similar patterns in kin and non-kin odor discrimination and investigation tasks^44^. Further, Belding’s ground squirrels, *Urocitellus beldingi*, also discriminate between novel kin, familiar kin, novel kin, and novel non-kin^42,43^. This trend in discriminating across components of conspecific identity suggests there are important components of familiarity and novelty that are separable.

It is yet unclear why kin status drove differences in the time spiny mice spent engaged in prosocial behavior. One possibility is that novel kin are particularly salient because they are less frequently encountered compared to novel non-kin members. Supporting this possibility, investigation accounted for most of the subjects’ prosocial behavior time. Recognition of novel kin in addition to familiar kin can optimize foraging behavior^56^, decrease stress^57^, and allow for nepotism^58^. Indeed, kin selection is commonly evoked to explain these benefits, as any genetic relation is a potential opportunity for shared genes to move to future generations^59,60^. It is no surprise, then, that many scientists consider kin recognition directly when discussing kin selection^18^, though social learning has also been suggested as an alternative mechanism fostering kin recognition^18,61,62^. Regardless, how kin relation alters other behaviors in spiny mice is an open question that future studies should aim to address.

### Pheromonal communication and bodily location of glands

Rodents produce informational odors and pheromones through glands located across their bodies. For example, mice scent mark with their urine^63^, the greater long-tailed hamster, *Tscheskia triton*, and golden hamster, *Mesocricetus auratus*, have left and right flank and midventral glands used for flank marking^64,65^, and Mongolian gerbils, *Meriones unguiculatus*, have harderian glands on their face^66^ and mid-ventral sebaceous glands for scent and pheromone release^67^. While spiny mice exhibit phenotype matching^19^, it is currently unknown where scent glands are located on spiny mice, but most rodents have glands on their face, flanks, and anogenital region (referred to here as “rear”) that emit olfactory and pheromonal cues. It is likely that individual glands provide specific cues that vary in salience to conspecifics based on their identity. In our study, male spiny mice investigated the flanks of familiar non-kin conspecifics more than their head and showed a statistical trend towards investigating novel kin members more than novel non-kin both overall and at their flanks. An alternative to olfactory cues, however, is whisker-to-whisker contact, often referred to as “social facial touch” seen in rodents, such as rats^68^. Social facial touch may provide additional information about a conspecific, such as their identity or their recent behavior. Any face investigation with familiar conspecifics in spiny mice may be due to similar motivations. However, more work is needed to locate scent glands on spiny mice and determine their effects on behavior as well as to differentiate head investigation for olfactory cues versus whisker communication.

### The LS and social discrimination

The LS is increasingly considered a critical region for regulating social behavior^35,40,41^, and was recently shown to be topographically mapped for responsiveness to kinship in Long Evans rats^36^. Rats had more non-kin responsive neurons within the dorsal LS and more kin responsive neurons in the ventral LS^36^. In contrast, we found that the spiny mice dorsal LS exhibited greater neural responsivity to novel kin compared to novel non-kin and that the LS did not distinguish between novel and familiar non-kin. These results run counter to those of Clemens et al.^36^ and suggest that there may be species differences in the functional organization of the LS. Additionally, our study controlled for familiarity, while the Long Evans rat study used familiar littermates and mothers as the kin stimuli and novel individuals as the non-kin stimuli. Furthermore, we examined IEG responses from freely behaving animals, which lack the temporal resolution of the single-cell patch clamping of head-fixed animals used in the Clemens et al.^36^ study. Based on these details, differences between our studies may instead reflect methodological discrepancies. Regardless, the dorsal LS appears to play a critical role in social recognition, particularly for distinguishing kinship status in spiny mice.

In our study, the novel kin condition was the only condition that showed both an increase in neural responsivity as well as an increase in prosocial behavior. While we cannot rule out that it was prosocial behavior rather than kin recognition that drove greater neural responses within the dorsal LS, we found no correlation between dorsal LS neural responses and prosocial behavior, which would be expected if prosocial behavior was the primary driver of the differences in neural response. Additionally, because Clemens et al.^36^ found that cells within the dorsal LS distinguish between kin and non-kin, it is likely that our findings in spiny mice also reflect processing of kinship status rather than greater prosocial responses. Further studies, ideally neuro-manipulative in nature, are required to determine the direct role the dorsal LS and connected regions play in kin recognition.

Our results also show distinct differences in responsiveness to kinship between the dorsal and ventral LS, as well as differences in how neural responses in these subregions of the LS relate to behavior. Interestingly, neural responses in the ventral LS positively correlated specifically with investigation in male spiny mice that interacted with novel conspecifics (both kin and non-kin), but not familiar conspecifics. This brain-behavior relationship suggests that the ventral LS may be particularly important for processing tactile and olfactory information of novel individuals. Although ventral LS neurons may be important for either promoting social investigation or processing novel social information, it is possible that we did not observe global ventral LS neural responsivity differences between animals exposed to novel vs. familiar conspecifics because other social behaviors directed toward the conspecifics did not significantly differ (i.e. we found no differences in prosocial or aggressive behaviors). These findings add to the growing literature depicting the LS as a highly heterogeneous region made up of several subregions. Indeed, while the LS is mostly GABAergic^32,69^, there are robust structural and genetic differences between the various subregions^32,40,41,69,70^. These differences have already been shown to have functional consequences outside of social recognition. For example, a small population of neurons within the LS of C57BL/6 J mice receives oxytocin from the VTA and promotes aggressive behavior^32^, and ventral LS mGlu2/3 receptors promote stress resilience in male C57BL/6 J mice^71^. Furthermore, subdivision of the LS is not unique to mammals; neurochemical examination of the LS in finches and waxbills yielded multiple chemoarchitectonic subzones within the LS, with neurochemical profiles suggesting the LS is involved in an array of social behaviors. Indeed, studies have shown that the LS regulates prosocial behavior^72–75^ as well as aggression^32,40,76,77^. Together, these findings highlight the importance of exploring how subregions and cell types within the LS respond to different social contexts rather than treating the LS as a monolithic structure.

### Conclusion

In the present study, we demonstrated that spiny mice modify their behavior based on the familiarity and kinship status of individuals. Spiny mice exhibit strong social recognition abilities in a novel group setting and preferentially affiliate with novel kin over novel non-kin or familiar kin. This affiliative preference in a group interaction coupled with the exhibition of more prosociality with novel kin over novel non-kin during dyadic interactions suggests a strong bias toward kin even though kin and non-kin maintain positive social relationships in this species. We further showed that the LS distinguishes between kinship, but not familiarity, status, demonstrating that there are distinct types of social recognition that are differentially represented in the brain. Together, our study highlights the complex dynamics of social recognition in the communally breeding spiny mouse and lays a foundation for future studies that may seek to identify the neural underpinnings of novel vs. familiar social discrimination or explore the involvement of motivational systems in social recognition and preferences.

## Materials and methods

### Animals

Forty adult male *Acomys cahirinus* (post-natal day (PND) 95-220) were used for behavioral testing and 32 adult male *A. cahirinus* (PND 60-512) were used for immediate early gene (IEG) studies. All animals were obtained from our breeding colony; breeders were from the captive-bred colony of Dr. Ashley W. Seifert (University of Kentucky). Dr. Seifert’s colony has been maintained for 10 + years. All animals were group-housed (2–4) in either a standard rat polycarbonate cage (40.64 × 20.32 × 20.32 cm) or a larger two-level polycarbonate cage (32 × 38 × 40 cm) lined with Sani-Chips bedding. Animals were provided with nesting material, rodent igloos, and shepherd shacks and were able to obtain food and water ad libitum. Spiny mice were kept on a 14-h light: 10-h dark cycle with an ambient temperature of 24 ± 2 °C. All procedures were approved by the Institutional Animal Care and Use Committee of Emory University (PROTO201900126). All methods were conducted in accordance with relevant ARRIVE guidelines and regulations. All methods were performed in accordance with the relevant guidelines and regulations. Due to a lack of female spiny mouse availability in our colony, we were only able to conduct this study in males. Future studies will be needed to examine the influence of novelty/familiarity and kinship on spiny mouse female behavior.

### Experimental design

To identify if male spiny mice recognize and behave differently with individuals based on novelty and kinship status, we ran 1 cohort of males through a group social interaction test and a different cohort of males through dyadic social interaction IEG tests. Stimulus mice were color coded with a small amount of animal-safe marker for unique identification. All tests were video recorded using Sony Handycam HDR-CX405 1080p Camcorders (Sony) for subsequent scoring using Behavioral Observation Research Interactive Software^78^ or a modified hand-scoring method. At the end of each group interaction, the testing arena was cleaned with Virkon-S, followed by water and then towel dried (fresh towel each time) to eliminate odors from the previous group. All IEG tests were conducted in separate clean cages.

### Group social interaction

To determine if male spiny mice investigate and affiliate with different conspecific types at different rates in a dynamic social environment, 5 groups of 8 males (PND 80-200) were placed into a novel arena (58L × 120W × 60H cm) containing two transparent rodent igloos and were allowed to freely interact for 1 h. Each subject was familiar with only one other conspecific—their sibling/cagemate. 2 conspecifics were novel kin, and the remaining 4 animals were novel non-kin. Novel kin were at least 2 litters removed from the subject spiny mice. Time sampling behavioral observations were recorded for each subject every 5 min to obtain frequencies of social contact (i.e. investigation and positive affiliation (i.e. positive side-by-side contact and huddling)) with different conspecific types.

### Immediate early gene studies

To identify differences in the neural response of the LS based on conspecific identity, 32 male spiny mice (PND 60-512) underwent 1 of 2 IEG studies: (1) males engaged in a social interaction with a novel kin conspecific or a novel non-kin conspecific (kin vs non-kin IEG) or (2) males engaged in a social interaction with a novel non-kin conspecific or a familiar non-kin conspecific (novel vs familiar IEG). All novel non-kin conspecifics were at least 2 litters apart, ensuring they had never interacted prior to the IEG. Familiar non-kin stimulus animals were obtained by rehousing subjects in a large two-level cage with a divider installed. The cage divider was a Plexiglas barrier that contained 1 cm holes to allow for tactile and olfactory communication. Two subjects (i.e. siblings) were housed on one side, and 2 non-kin conspecifics were housed on the other side. Subjects and stimulus animals cohabitated with the divider installed for 7 days prior to undergoing the IEG study.

For the IEG tests, subjects were placed simultaneously into a standard rat polycarbonate cage (40.64 × 20.32 × 20.32 cm) with 1 of 3 possible conspecific types (novel kin, novel non-kin, familiar non-kin). Subjects interacted with the stimulus conspecific for 30 min before being transferred to a second, clean rat cage for an additional 30 min prior to undergoing a perfusion to capture Fos-ir + expression in response to the stimulus exposure. All interactions were recorded, and the first 5 min scored for prosocial (allogrooming, positive side-by-side contact, huddling, head investigation, flank investigation, and rear investigation), aggressive (biting, chasing, lunging, pinning, rearing, and aggressive side-by-side contact) and non-overt (all remaining time of scored recording) behaviors (Table S3). For social investigation, we analyzed behavior by bodily location (flank, head, or rear) as well as all bodily locations of investigation together. The two IEG tests (kin vs. non-kin and novel vs. familiar) were conducted separately, and thus were analyzed as separate tests.

### Histology and immunohistochemistry

At the end of both IEG tests, subjects were immediately euthanized by isoflurane overdose and were transcardially perfused with 0.1 M phosphate buffer saline (PBS) followed by 4% paraformaldehyde. Brains were extracted, post-fixed overnight in 4% paraformaldehyde, and underwent cryoprotection in 30% sucrose dissolved in PBS for 48 h. Brains were frozen in Tissue-Tek O.C.T. compound and stored at − 80 °C before sectioning coronally at 40 µm using a Leica cryostat, with every third section saved for use in the present study. Tissue sections were immunofluorescently stained for Fos (the protein of the immediate early gene *cFos;* Synaptic Systems rabbit c-fos 1:1000 dilution) and NeuN, a neuron-specific nucleus marker (Millipore mouse NeuN 2:1000 dilution).

### Neural quantification

**Figure 4:**
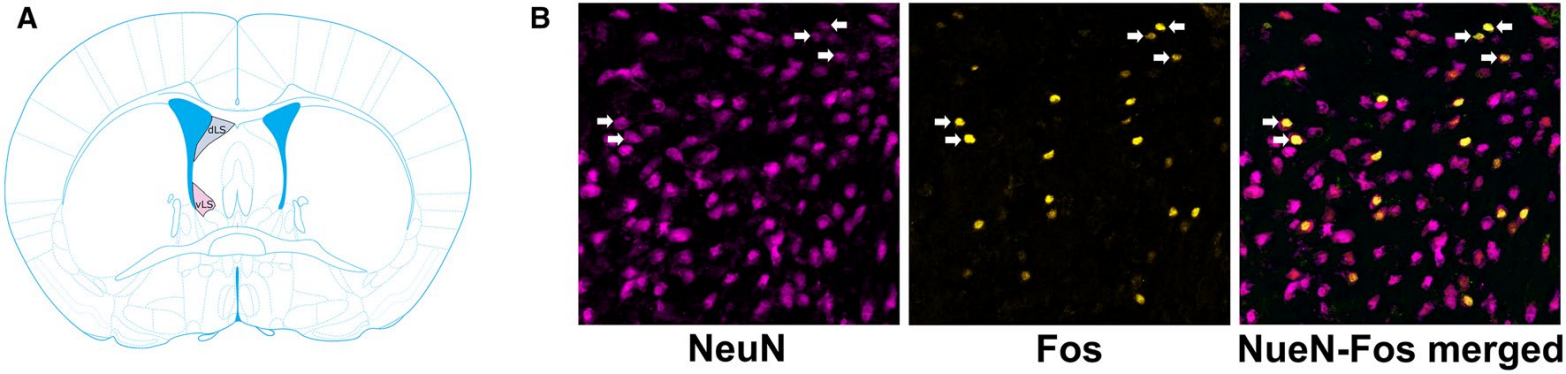
Dorsal and ventral lateral septum regions and representative images of NeuN-Fos colocalization. (**A**) Illustration of a mouse coronal section showing the subdivision of the dorsal (dLS; blue) and ventral (vLS; pink) lateral septum (LS) used for cell counts (image edited from Paxinos & Franklin^83^). (**B**) 40 × images in spiny mice of NeuN (left), Fos (center), and merged NeuN-Fos colocalization (Right). White arrows indicate colocalization.

Photomicrographs were obtained using a Zeiss AxioImager II microscope fitted with an apotome. For LS cell counts, we took 10 × images and quantified the total number of Fos-immunoreactive (-ir) and NeuN-ir cells and the number of NeuN-ir neurons that co-expressed Fos across 6 tissue sections for the novel vs familiar IEG and 7 sections for the kin vs non-kin IEG in both the dorsal and ventral LS (note that the tissue section number discrepancy was due to tissue availability, and that data from the 2 IEG studies are analyzed separately) (Fig. 4). These tissue sections spanned a-p coordinates of + 1.5 mm To + 1.78 mm from bregma and d-v coordinates of − 4.4 to − 3.0 mm from the top of cortex based on a recent stereotaxic atlas^79^ Note that hodological evidence of the division of the LS is lacking in spiny mice, however studies in numerous species have demonstrated that there is anatomical and functional subdivision of the LS with strong consistency in dorsal and ventral subdivision in rodents^80^. Thus, while we capture the dorsal and ventral portions of the LS of spiny mice, we acknowledge that future studies may further refine these subdivisions. FIJI^81^ was used to create standard ROIs for all dorsal and ventral LS images, and a cell profiler^82^ pipeline was created to automatically count fluorescent cells and nuclei and identify colocalized neurons. The values across all sections were summed and a percentage of the number of NeuN-ir cells that expressed Fos-ir across was used to account for individual differences in cell number. Tables S4 and S5 provide group averages for NeuN-ir cell counts. Significant statistical differences were identified between the dorsal and ventral LS and were thus analyzed separately.

### Statistical analysis

Behavioral measurements for each test were analyzed using SPSS 28 (IBM Analytics). For the group social interaction, investigation and affiliation frequencies were transformed to z-scores for each subject by conspecific type to account for the greater number of opportunities to interact with novel non-kin than novel or familiar kin. The use of parametric or non-parametric tests was based on the distribution of the data and Shapiro-wilks tests. Tests used include Poisson generalized linear mixed model (GLMM) with condition as a fixed factor and subject and group as random factors, general linear models (GLM) with condition and region (ventral versus dorsal) as fixed factors, Friedman’s tests, Mann–Whitney *U* tests, and Wilcoxon ranked-sum tests. All *post-hoc* pairwise comparisons were adjusted using either the Bonferroni, Sidak, or false discovery rate correction, depending on the statistical test used. The tests used for specific analyses are detailed in the Results. Pearson’s correlations were calculated between percentage of colocalization cell counts and behavioral measures. Outliers for each individual test were 3 standard deviations outside the mean and were removed from analyses. Effect sizes for normally distributed data were calculated and reported as Cohen’s *d*, whereas effect sizes for nonparametric analyses were reported as r where $r= \frac{z}{\sqrt{N}}$*.*

### Supplementary Information

Supplementary Information.

## Data Availability

Data are available upon request from the corresponding author.
