# Author Correction: Biased brain and behavioral responses towards kin in males of a communally breeding species

**DOI:** 10.1038/s41598-023-47250-1

**Published:** 2023-11-16

**Authors:** Brandon A. Fricker, Deborah Ho, Ashley W. Seifert, Aubrey M. Kelly

**Affiliations:** 1 Department of Psychology, Emory University, 36 Eagle Row, Atlanta, GA 30322 USA; 2 Department of Biology, University of Kentucky, 101 Morgan Building, Lexington, KY 40506 USA

Correction to: *Scientific Reports* https://doi.org/10.1038/s41598-023-44257-6, published online 09 October 2023

The original version of this Article contained an error in the Materials and methods section, under the subheading ‘Animals’.

“Fourteen adult male *Acomys cahirinus* (post-natal day (PND) 95-220) were used for behavioral testing and 32 adult male *A. cahirinus* (PND 60-512) were used for immediate early gene (IEG) studies.”

now reads:

“Forty adult male *Acomys cahirinus* (post-natal day (PND) 95-220) were used for behavioral testing and 32 adult male *A. cahirinus* (PND 60-512) were used for immediate early gene (IEG) studies.”

The original Article has been corrected.

