# Supplementary material for: Biased brain and behavioral responses towards kin in males of a communally breeding species
Source: Sci Rep. 2023 Oct 9;13:17040. doi: 10.1038/s41598-023-44257-6 (PMC10562393; doi:10.1038/s41598-023-44257-6)
Supplement: Supplementary file 1 — Supplementary Information. [file 41598_2023_44257_MOESM1_ESM.docx]

**Supplemental Materials**

**
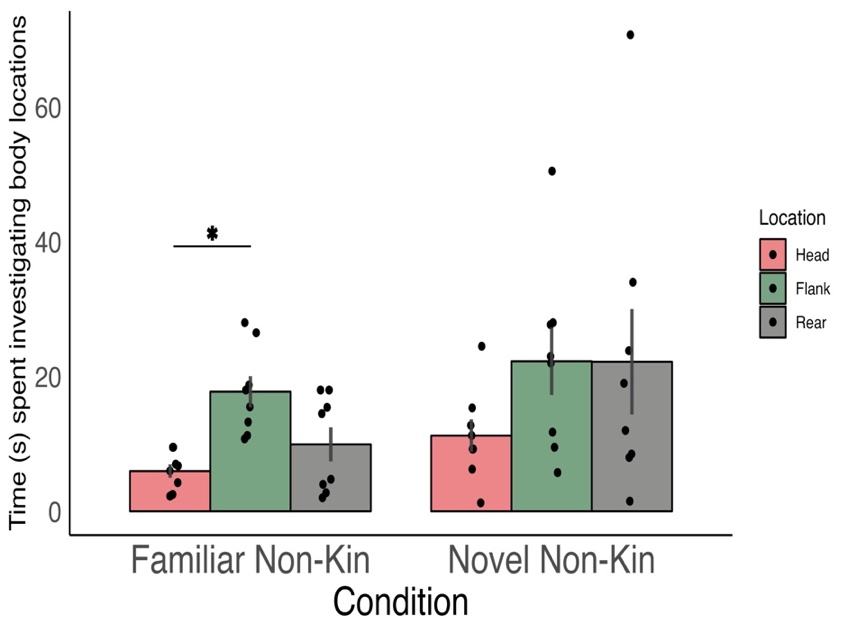
**

**Fig. S1. Male spiny mice differentially investigated parts of the body of familiar but not novel non-kin conspecifics.** Male spiny mice mean ($\pm$ SEM) time in seconds (s) for investigating the flank (green), head (pink), and rear (grey) of familiar (left) and novel (right) non-kin conspecifics. Male spiny mice significantly investigated the flank more than the head of familiar non-kin but did not differentiate the bodily location of investigation for novel non-kin. Dots represent individual data. * Indicate P ≤ 0.05.


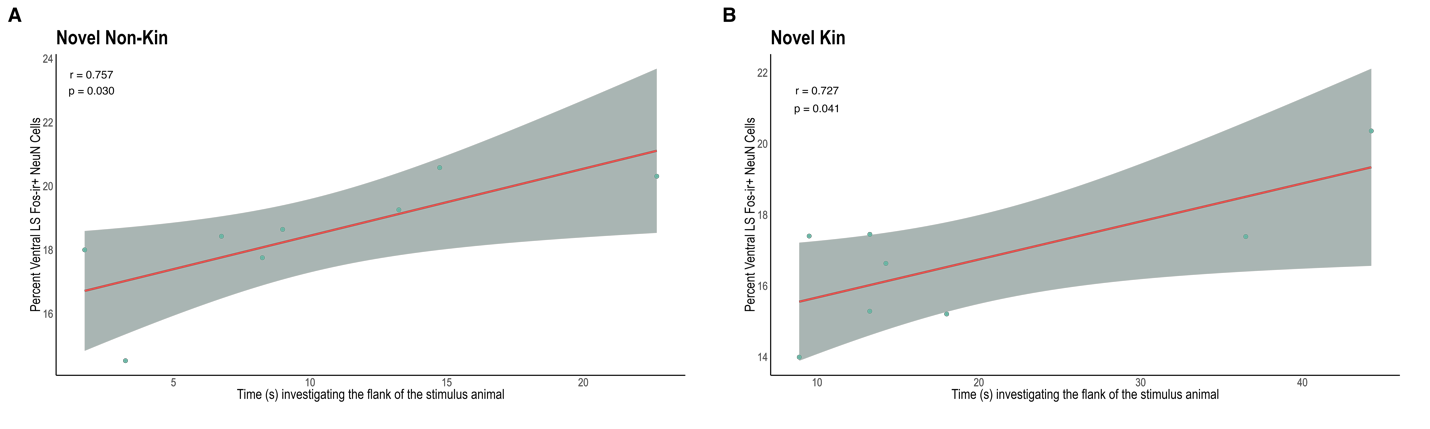


**Fig S2. Neural responses in the ventral lateral septum correlate with investigation of novel kin and non-kin.** Correlations (± 95% CI in green) between the percentage of NeuN-Fos colocalized cells in the ventral lateral septum (LS) and time in seconds (s) engaged in **(A)** flank investigation of novel non-kin and **(B)** flank investigation of novel kin. Dots represent individual data.

**Table S1.** Breakdown of behavior during the novel non-kin vs familiar non-kin social interaction immediate early gene test.

| Conspecific | Comparison | Test | χ2/Z | P | r |
| --- | --- | --- | --- | --- | --- |
| Novel Non-Kin | Overall | Friedman’s test | 16 | <0.01* |  |
|  | Overt Prosocial***** vs Overt Aggression | Wilcoxon | -2.521 | 0.012* | 0.88 |
|  | Overt Prosocial vs Non-Overt***** | Wilcoxon | -2.521 | 0.012* | 0.88 |
|  | Overt Aggression vs Non-Overt***** | Wilcoxon | -2.521 | 0.012* | 0.88 |
| Familiar Non-Kin | Overall | Friedman’s test | 16 | <0.01* |  |
|  | Overt Prosocial***** vs Overt Aggression | Wilcoxon | -2.521 | 0.012* | 0.88 |
|  | Overt Prosocial vs Non-Overt* | Wilcoxon | -2.521 | 0.012* | 0.88 |
|  | Overt Aggression vs Non-Overt***** | Wilcoxon | -2.521 | 0.012* | 0.88 |

Note: under comparison * means greater time.

**Table S2.** Breakdown of behavior during the novel kin vs novel non-kin social interaction immediate early gene test.

| Conspecific | Comparison | Test | χ2/Z | P | r |
| --- | --- | --- | --- | --- | --- |
| Novel Non-Kin | Overall | Friedman’s test | 16 | <0.01* |  |
|  | Overt Prosocial***** vs Overt Aggression | Wilcoxon | -2.521 | 0.012* | 0.88 |
|  | Overt Prosocial vs Non-Overt***** | Wilcoxon | -2.521 | 0.012* | 0.88 |
|  | Overt Aggression vs Non-Overt***** | Wilcoxon | -2.521 | 0.012* | 0.88 |
| Novel Kin | Overall | Friedman’s test | 16 | <0.01* |  |
|  | Overt Prosocial***** vs Overt Aggression | Wilcoxon | -2.521 | 0.012* | 0.88 |
|  | Overt Prosocial vs Non-Overt* | Wilcoxon | -2.521 | 0.012* | 0.88 |
|  | Overt Aggression vs Non-Overt***** | Wilcoxon | -2.521 | 0.012* | 0.88 |

Note: under comparison * means greater time.

**Table S3.** Ethogram for immediate early gene dyad social interactions.

|  | Behavior | Description |
| --- | --- | --- |
| Prosocial | Head Investigation | Subject sniffing or positively investigating the stimulus animal’s head. |
|  | Flank Investigation | Subject sniffing or positively investigating the stimulus animal’s flanks. |
|  | Rear Investigation | Subject sniffing or positively investigating the stimulus animal’s rear. |
|  | Allogrooming | Subject grooms the stimulus animal. |
|  | Huddling | Subject and stimulus are either touching flanks or criss-crossed on top of each other. |
|  | Positive Side-by-Side Contact | Subject showing positive, prosocial contact side-by-side with the stimulus that is not specifically huddling. |
| Aggressive | Biting | Subject biting at the stimulus animal, mouth making contact with the stimulus animal's body. |
|  | Chasing | Subject aggressively chasing the stimulus animal. Initiator is chaser for entire event. |
|  | Pinning | Pinning the stimulus down |
|  | Rearing | Subject rearing up on hind paws for either offense or defense. |
|  | Aggressive Side-by-Side Contact | Subject and stimulus are touching flanks but in an aggressive manner. May be between aggression bouts. |
| Non-overt | All behavior instances not included in Prosocial or Aggressive. | Subject is not making contact with or exhibiting behavior direct toward stimulus animal. |

**Table S4.** Breakdown of NeuN and Fos+ NeuN cells by Conspecific condition and LS subregion for the Novel vs Familiar Non-kin IEG.

| Conspecific | Subregion of LS | Average Fos+ NeuN | Average NeuN | % of Fos+ NeuN Cells |
| --- | --- | --- | --- | --- |
| **Novel Non-Kin** | Dorsal | 127.17 | 712.31 | 18.12 |
|  | Ventral | 159.56 | 911.875 | 17.56 |
| **Familiar Non-Kin** | Dorsal | 124.73 | 789.71 | 15.73 |
|  | Ventral | 104.94 | 661.90 | 15.91 |

Note: Values are rounded to the second decimal place based on descriptive analysis output.

**Table S5.** Breakdown of NeuN and Fos+ NeuN cells by Conspecific condition and LS subregion for the Novel Non-Kin vs Novel Kin IEG.

| Conspecific | Subregion of LS | Average Fos+ NeuN | Average NeuN | % of Fos+ NeuN Cells |
| --- | --- | --- | --- | --- |
| **Novel Non-Kin** | Dorsal | 138.86 | 635.54 | 21.88 |
|  | Ventral | 116.43 | 630.46 | 18.44 |
| **Novel Kin** | Dorsal | 178.70 | 631.73 | 27.65 |
|  | Ventral | 114.75 | 689.09 | 16.72 |

Note: Values are rounded to the second decimal place based on descriptive analysis output.
